# A Digital Patient Decision Aid to Increase Sexually Transmitted Infection Testing in the Emergency Department: Protocol for a Pilot Randomized Controlled Trial of the STIckER (STI Check in the Emergency Room) Study

**DOI:** 10.2196/67103

**Published:** 2025-12-02

**Authors:** Jason Zucker, Delivette Castor, Marc A Probst, Talia Adler, Lisa-Pierre Tchoungui, Jaciara De Souza, Christina Franqui, Lauren Chernick

**Affiliations:** 1 Division of Infectious Diseases, Department of Medicine, Columbia University Medical Center, 622 West 168th street, New York, NY, 10032, United States, 1 2017236637; 2 Department of Emergency Medicine, Columbia University Medical Center, New York City, NY, United States

**Keywords:** adolescent sexual health, emergency department STI testing, sexually transmitted infection, youth, mHealth, mobile apps, digital health, STI

## Abstract

**Background:**

Rising rates of sexually transmitted infections (STIs) among adolescents and young adults are concerning. Emergency department (ED) visits offer a unique opportunity to increase STI testing. However, providers in the ED may not have the time or self-efficacy to screen patients, and patients may be unable to initiate discussion on their STI care needs. A patient decision aid may improve the effectiveness and efficiency of the ED encounter for STI testing.

**Objective:**

This study aims to pilot STI Check in the Emergency Room (STIckER) app, a shared decision-making aid for urinary, oral, and rectal gonorrhea and chlamydia testing among adolescents and young adults, and assess STIckER’s efficacy and implementation outcomes in an adult and pediatric ED.

**Methods:**

We are conducting a parallel-group, two-arm provider-randomized controlled pilot trial in the adult and pediatric EDs at an urban academic medical center. Fifty providers were randomized 1:1 to intervention (trained on STIckER) or control (standard of care). Adolescents and young adults aged 14‐24 years, English-speaking, and sexually active within the past 6 months are recruited during eligible shifts and assigned to the study arm of their provider. The standard of care comparator consists of usual provider-initiated STI discussions and testing without access to the decision aid; control participants receive a QR code directing them to a simple landing page encouraging them to ask their provider about STI testing. The planned enrollment target was 44 providers and 140 adolescent and young adult participants. Primary outcome is preliminary efficacy, defined as the proportion of adolescents and young adults receiving gonorrhea and chlamydia testing based on electronic medical record (EMR) review. Secondary outcomes include extragenital testing uptake, STI positivity rates, decisional conflict, and perceived level of shared decision-making. Implementation outcomes include feasibility, acceptability, appropriateness (using acceptability of intervention measure, intervention appropriateness measure, and feasibility of intervention measure), and provider self-efficacy. Data will be analyzed using descriptive statistics and regression models, with mixed-effects models applied if clustering by provider is detected. Exploratory analyses will examine differences by patient age, gender, and sexual identity, and provider characteristics.

**Results:**

Between September 2023 and July 2025, we completed recruitment of 44 providers and 139 adolescent and young adult participants across both adult and pediatric EDs. Enrollment targets were met, with high fidelity to intervention delivery and strong rates of survey completion by both patients and providers. Final data cleaning and analyses are underway, and primary results are expected to be available in late 2025.

**Conclusions:**

This pilot trial will provide critical data on the feasibility, acceptability, and preliminary efficacy of a digital SDM tool to increase STI testing in the ED. Findings will inform the design of a future multisite trial and the potential for scaling STIckER to support equitable, patient-centered STI screening across diverse clinical settings.

## Introduction

Sexually transmitted infections (STIs) pose a significant and growing public health challenge in the United States. In 2022, more than 2.5 million cases of syphilis, gonorrhea, and chlamydia were reported nationally [1]. Gonorrhea and chlamydia require comprehensive screening to detect infections across multiple anatomic sites. Studies have shown that failure to conduct complete screening, including extragenital screening of oropharyngeal and anorectal sites, can lead to underdiagnosis. In fact, research suggests that if only urogenital screening is performed, more than one-third of infections may be missed [2].

Despite this, comprehensive gonorrhea and chlamydia screening, including 3-site screening, remains underused. Less than 5% of emergency department (ED) patients presenting with symptoms suggestive of extragenital infection undergo appropriate screening [3,4]. In outpatient settings, extragenital STIs are also prevalent but often undetected, with studies reporting prevalence rates as high as 77% among females and 22% among heterosexual males [3]. These data underscore the critical need for improved screening strategies that support multisite screening, particularly in settings where vulnerable individuals are likely to present.

The ED represents a vital yet underused venue for STI screening. Each year, over 19 million adolescents and young adults seek care in EDs, many of whom rely on these settings as their primary source of health care [4,5]. However, data suggest that the prevalence of STIs among adolescents and young adults in EDs ranges from 1.8% to 25%, representing substantial unmet need. Despite the potential for EDs to serve as access points for STI care, providers face substantial challenges to routine screening implementation. Time constraints, competing clinical priorities, lack of confidence in sexual history-taking, and discomfort discussing sexual health with patients all limit the integration of STI screening into ED workflows [6-9]. These limitations are especially problematic when caring for adolescent and young adult patients, who may face additional barriers including stigma, concerns around confidentiality, lack of knowledge about STI risks, and discomfort disclosing sensitive information in a high-acuity setting [10].

Novel strategies are needed to increase HIV and syphilis screening in the ED setting. While universal STI genitourinary testing minimizes patient stigma and provider bias, targeted testing recognizes the limited ED resources [11-13]. Furthermore, despite recommendations for universal opt-out HIV testing, fewer than 1% of ED patients are routinely screened. Researchers are currently focusing on clinical decision support (CDS) tools incorporated into the EMR [14]. While CDS tools are promising, they rely on each EMR system, thus leaving a gap in the care provided and missed opportunities for testing.

Shared decision-making (SDM) has been recognized as a valuable strategy for promoting patient-centered care, including for sexual health. However, implementing SDM in the fast-paced ED setting is difficult due to limited visit time, competing priorities, and a lack of standardized tools. Digital decision aids can help address these challenges by delivering tailored health education, collecting patient preferences, and streamlining communication with providers.

While decision aids have shown benefit in improving patient engagement and satisfaction in other ED contexts, including for decisions around chest pain, pain management, and syncope, there is limited work applying this approach to STI care [15-19]. No existing digital tool has been developed to support comprehensive gonorrhea and chlamydia screening, including extragenital screening, in the ED setting using an SDM framework.

To address these gaps, we developed STIckER (STI Check in the Emergency Room), a mobile, web-based decision aid designed to promote SDM between providers and adolescent and young adult patients in the ED. STIckER aims to support 3-site gonorrhea and chlamydia screening through a patient-centered approach grounded in evidence and stakeholder input. The tool guides users through an educational module, a sexual health risk assessment, and tailored screening recommendations, culminating in a provider-facing display that facilitates brief but informed shared decision-making. By front-loading STI education and risk assessment, STIckER reduces the time providers must spend initiating sensitive discussions and provides a standardized way to present testing options. Simultaneously, the app empowers adolescents and young adults by normalizing STI screening, increasing awareness of extragenital testing, and encouraging them to articulate their preferences. These features are designed to foster confidence in requesting testing and to facilitate efficient, values-based shared decision-making during time-limited ED encounters. STIckER was developed through an iterative process involving input from 19 adolescent and young adult patients and 7 ED providers [20]. By targeting known barriers to STI screening and supporting both patients and providers, STIckER aims to improve the delivery of equitable and complete STI care in the ED environment.

## Methods

### Trial Design

This parallel group, 2-arm, provider-level randomized controlled trial (RCT) was designed as a pilot evaluation to assess the preliminary effectiveness and implementation process of the STIckER app in an adult and pediatric emergency department (ED). The study followed CONSORT-eHEALTH guidelines V1.6 for reporting electronic and mobile health (mHealth) interventions.

### Participants

#### Provider Eligibility Criteria

A subset of health care providers (HCPs) was invited to participate. Eligible HCPs were attending physicians, residents, or nurse practitioners serving in the adult and pediatric EDs at NewYork-Presbyterian/Columbia University Irving Medical Center (NYP/CUIMC), a larger urban academic medical center in the Northern Manhattan section of New York City.

#### Patient Eligibility Criteria

Eligible patients were those being seen by an enrolled HCP, between ages 14 and 24 years, sexually active within the past 6 months (per self-report), able to speak English, and assigned a triage level of 3, 4, or 5. These triage levels indicate that the patient was not critically ill. Medical records were reviewed to identify potential subjects. Exclusion criteria include severe illness, cognitive impairment, or inability to speak English.

#### Providers Recruitment

HCPs were recruited via department meetings, email, and in-person outreach. All HCPs were educated about the study and viewed a recorded presentation on shared decision making and STI testing. HCPs who volunteered for enrollment provided written informed consent.

#### Participant Recruitment

Patients are recruited during enrollment blocks, which correspond to the ED shifts of enrolled HCPs. After the patient was assessed by an HCP, a research team member approached eligible patients, introduced themselves, and presented the study without the HCP. Not all shifts will be covered, and providers were not informed of which shifts were sampled for enrollment. A research team member confirmed eligibility and obtained written informed consent from each adolescent and young adult and a waiver of parental consent for participants aged 18 years and younger, similar to other sexual health ED-based studies.

#### Randomization and Allocation

To avoid contamination, 50 HCPs were randomized 1:1 to the intervention (STIckER) or control arm. The allocation sequence was computer-generated by the study epidemiologist using a simple randomization method with stratification by location (adult vs pediatric ED). The randomization sequence was implemented by a study coordinator using a centralized allocation system. The assignment was not concealed from investigators. Randomization health care provider participant (HCP-P) in the intervention arm received 1:1 training with a research assistant on the STIckER app. If a participating provider left the ED before the end of the study period, a new provider randomized to the same study arm was invited to participate.

### Intervention

Adolescents and young adults between the ages of 15 and 24 years attending the ED during the time that an HCP trained on STIckER was on service would scan the STIckER app before seeing the provider. patients as study participants (HCP-Ps and adolescent and young adult participants).

Adolescent and young adult participants are assigned to the arm of the provider to whom they are assigned. Adolescent and young adult participants with intervention-arm providers will scan a QR code with their personal mobile device, linking them to the web-based STIckER app. They will interact with the app, completing a sexual health assessment and indicating their personal values and preferences before being led to a final infographic to be shown to the HCP-P. This infographic will alert the provider to both the adolescent and young adult participants’ need for oral, rectal, or urinary STI testing and their preference for testing.

Adolescent and young adult participants with control-arm providers will also scan a QR code with their personal mobile device, but it will lead them to a landing page with a prompt informing them they can ask their provider about STI testing. They will not have access to the digital tools and shared decision-making modules within the STIckER app.

All adolescent and young adult participants are followed throughout the ED visit. Adolescent and young adult participants will complete questionnaires at enrollment and upon ED discharge. These questionnaires comprise a comprehensive set of predominantly validated instruments and will capture sociodemographic characteristics, sexual behavior, substance use, perceived HIV risk, patient satisfaction, and decision-making quality. HCP-P will also complete a questionnaire after each adolescent and young adult visit and an end-of-study questionnaire focused on STIckeR implementation.

### STIckER Web-Based App

#### Control Participants

Participants in the control arm will scan a QR code and receive a statement reminding them they can always ask for STI testing.

#### Intervention Participants

**Figure 1.**
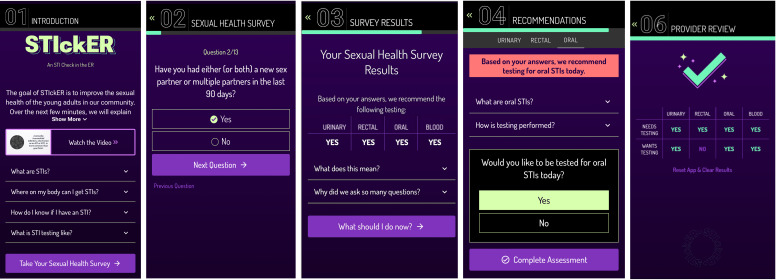
Overview of the STIckER (STI Check in the Emergency Room) web-based application modules. STIckER is available online, and this version was frozen during the study [21]. Panel A: Educational module with introductory video and supplemental information about sexually transmitted infection (STI) testing. Panel B: Sexual health risk assessment stratifying participants by need for genitourinary, oral, and rectal testing. Panel C: Shared decision-making modules, including values clarification and opt-in/opt-out testing choices. Panel D: Provider-facing infographic summarizing participant testing needs and preferences, revealed by the provider via touch activation.

Patient participants in the intervention arm will proceed through the following 5 modules (see Figure 1):

- STI education**:** STIckER starts with a 1:06-minute video describing the importance of STI testing, followed by additional information about STIs and testing for STIs (see Figure 1).
- Sexual health assessment: Participants complete a 13-question assessment to assess their vulnerability to STIs. This assessment categorizes participants into high, medium, or low need for testing and generates recommendations for genitourinary, rectal, and oral gonorrhea and chlamydia testing at the time of ED visit (see Figure 1).
- Decision-making and shared-decision-making modules: Participants are taken through each recommendation with additional education and the option to opt in or opt out of testing (see Figure 1, panel C). Participants who are “intermediate need” for needing testing at that visit are taken to an additional shared decision-making module that accounts for individual values and preferences (see Figure 1).
- Provider display: STIckER ends with a hidden display uncovered when the provider places their finger over the STIckER logo to reveal it. This reveals testing recommendations and patient choices for oral, rectal, and urinary testing (see Figure 1).

### Clinical Setting

This study was conducted in the adult and pediatric emergency departments of NewYork-Presbyterian/Columbia University Irving Medical Center, located in Northern Manhattan. These EDs collectively see more than 150,000 patient visits annually and serve a predominantly Hispanic patient population, with over 25% of patients living below the federal poverty line. The EDs provide safety-net care to many adolescents and young adults who lack consistent access to primary care. The high volume, demographic diversity, and prevalence of structural barriers to care in this setting make it particularly well-suited for piloting an intervention designed to support STI screening among underserved adolescent and young adult populations.

### Participants and Recruitment

The target enrollment for this study is 44 HCP-Ps and 140 adolescent and young adult participants.

For HCP-Ps, all ED attending physicians and physician assistants employed in the EDs, who work more than 5 shifts per month, are eligible. ED providers were recruited via a department-wide email request for participants. STIckER research coordinators will explain the study to interested HCPs and obtain written informed consent. HCP-Ps were selected to purposely sample half pediatric and adult ED providers with a range of professional experience.

For adolescents and young adult participants, all patients aged between 14 and 24 years, regardless of chief complaint, are eligible for screening. Adolescents and young adults aged between 14 and 24 years who self-reported sexual activity within the last 6 months and were able to speak English are eligible for enrollment. Participants who were in foster care, wards of the state, or in police custody, as well as those who were cognitively impaired, exhibited aggressive behavior, or were critically ill, as determined by their attending medical provider, were not eligible.

Research coordinators will screen ED patients during clinical hours by the enrolled HCP-Ps. Those times typically consisted of Monday-Friday, 8 AM-10 PM, with limited weekend enrollment. First, information to determine eligibility was gathered from the EMR (ie, age). Next, if a patient was deemed eligible to be assessed further, a research coordinator approached the patient to confirm criteria such as sexual activity and interest in the study. Eligible participants were enrolled with written informed consent. For participants aged between 14 and 17 years, we will obtain a waiver of parental consent and a 2-person verification of agreement to participate, such as the research coordinator and the physician attending the ED. Participants received a US $10 pay card at enrollment.

### Study Assessments

#### HCP-P Study Assessments

At enrollment, enrolled HCP-Ps will complete a basic demographic questionnaire. (age, race, ethnicity, sex at birth, gender identity, years since completing training, and type of clinical training completed).

The exit survey will be conducted immediately after a visit with an adolescent and young adult participant; the HCP-P will complete an exit survey.

HCP-Ps randomized to the control arm get questions on whether STI testing was discussed (“Did you and your patient discuss STI testing”), if the discussion was appropriate (Likert scale of 1‐5, from “completely appropriate” to “completely inappropriate”), if STI testing was ordered, and if STI testing was ordered what level of shared decision-making involvement occurred using the Control Preferences Scale (Likert scale of 1 to 5, from “I made the decision on my own” to “the patient made the decision on their own”) [22].

HCP-Ps randomized to the intervention will get the same questions on whether STI testing was discussed, if the discussion was appropriate, if STI testing was ordered, and if STI testing was ordered, what level of shared decision-making involvement occurred using the control preferences scale. They will also complete validated questions on implementation (acceptability of intervention measure [AIM], intervention appropriate measure [IAM], and feasibility of intervention measure [FIM]) [23].

The postsurvey will be conducted after completing their participation in the STIckER study (a maximum of 6 adolescent and young adult participants or the study completing overall enrollment), HCP-P will complete a postintervention questionnaire.

HCP-Ps, randomized to the control arm, will complete a self-efficacy questionnaire to measure their clinical communication skills [24].

HCP-Ps randomized to the intervention arm will complete the self-efficacy questionnaire measuring their clinical communication skills, a Perceived Characteristics of Intervention Scale (a 20-item assessment measure that covers 9 subconstructs: relative advantage, compatibility, complexity, trialability, observability, potential for reinvention, task issue, nature of knowledge, and technical support), a question on how easy it was To use the screen the participant showed them (Likert scale of 1 to 5, from “Complicated” to “Able to use the app immediately”), and open-ended questions about whether the STIckER app was acceptable and what changes they would like to see made [25].

#### Adolescent and Young Adult Participants Study Assessments

#### Enrollment

At enrollment, enrolled adolescent and young adult participants will complete a basic questionnaire to collect data on age, race, ethnicity, sex at birth, gender identity, sexual preferences, the reason for the ED visit, if they had a primary care visit in the past year, and if they had a visit, did they receive an HIV or STI testing.

#### STIckER

The adolescent and young adult participants, randomized to the intervention, will complete the STIckER web-based app, which includes a 13-question sexual health assessment. This includes a validated 6-question survey of age and STI-related behaviors, as well as locally developed questions about types of sexual activity engaged in the previous 3 months, and if they had received gonorrhea or chlamydia testing in the previous 30 days [26,27].

#### Exit Survey

Immediately after a visit with an HCP-P, the adolescent and young adult participants will complete an exit survey.

The adolescent and young adult participants randomized to the control arm will complete questions on whether STI testing was discussed (“Did you and your patient discuss STI testing”), if the discussion was appropriate (Likert scale of 1‐5, from “completely appropriate” to “completely inappropriate”), if STI testing was ordered, and if STI testing was ordered, then what level of shared decision-making involvement occurred using the control preferences scale. The Control Preferences Scale is used to assess the extent to which patients perceived that the decision about STI testing was shared between themselves and their provider. It captures patient-reported involvement in the decision-making process by asking who took the lead in making the decision. While it does not measure specific components, such as depth of discussion or mutual agreement, it is a validated and widely used measure of perceived shared decision-making.

They will also complete 4 questions on the amount of information received, clarity of information received, helpfulness of information received, and if they would recommend that information to others; 9 items from the decisional-conflict scale on uncertainty. They will complete 2 items, providing a rating of the ED (0 to 10) and if they would recommend the ED to family or friends. Finally, they will complete the 13-question sexual health assessment that was included in STIckER.

The adolescent and young adult participants randomized to the intervention will complete questions on whether STI testing was discussed (“Did you and your patient discuss STI testing”), if the discussion was appropriate (Likert scale of 1‐5, from “completely appropriate” to “completely inappropriate”), if STI testing was ordered, and if STI testing was ordered, then what level of shared decision-making involvement occurred using the control preferences scale. They will also complete 4 questions on the amount of information received, clarity of information received, helpfulness of information received, and if they would recommend that information to others; 9 items from the decisional-conflict scale on uncertainty [28]. They will complete 2 items, providing a rating of the ED (0 to 10) and if they would recommend the ED to family or friends. Finally, they will complete the AIM, IAM, and FIM implementation measures.

### Outcomes

#### Primary Outcome

The primary outcome of preliminary efficacy is the difference in the proportion of sexually active adolescents and young adults tested for Neisseria gonorrhoeae or chlamydia trachomatis between the 2 arms based on the EMR review. We selected preliminary efficacy as the primary outcome because the trial was designed and powered to provide an effect size estimate that would inform the design and sample size calculations for a future multisite trial. While many pilot studies emphasize feasibility and acceptability as primary outcomes, in this single-site trial, we aimed to generate robust preliminary data on testing uptake to guide scale-up and ensure generalizability across diverse ED settings.

#### Secondary Outcomes

Secondary efficacy outcomes include rates of extragenital Neisseria gonorrhoeae or chlamydia trachomatis testing, overall and site-specific STI positivity rate, decisional conflict, and level of shared decision-making. Secondary implementation outcomes include feasibility (FIM), acceptability (AIM), appropriateness (IAM), and personal traits and attitudes toward STI screening.

### Statistical Analysis

We will summarize efficacy and implementation outcomes using standard descriptive statistics for continuous and categorical variables. We will conduct bivariate analyses to identify balance in the random allocation of the intervention across providers and patient characteristics.

#### Preliminary Efficacy

An intent-to-treat analysis of all enrolled patients will be conducted according to random assignment of the intervention to estimate the effect of STIckER on STI testing. We will conduct a mixed-effects generalized linear model (GLM) with the logit link function to look at the effect of STIckER on STI testing, if there is a clustering effect by provider, or logistic regression if there is no provider effect. To determine whether there is a provider effect, we will assess the intraclass correlation coefficient (ICC) for the primary outcome using a null (empty) mixed-effects model with provider as a random effect. If the ICC is greater than 0.01, we will proceed with mixed-effects modeling; otherwise, we will use standard logistic regression. For other dichotomous outcomes, we will use the same analytic approach (GLM) with the logit link function if there is a provider effect or logistic regression if there is no provider effect.

#### Secondary Efficacy and Implementation Outcomes

If there is a provider-level effect, we will use mixed-effect GLM to look at the association of STIckER on the continuous outcomes, such as implementation scales, and linear or logistic regression if there is no provider effect. If needed, we will also conduct sensitivity analyses to explore the effect of spillover, misclassification, missing data, or any other deviation from the study procedures that would affect efficacy or implementation outcomes.

#### Exploratory and Subanalyses

Additional exploratory analyses will examine differences in the primary and secondary outcomes among adolescent and young adult patients by age (older vs younger), gender, sexual identity, and other covariates of interest. Analyses of providers will include exploring differences by gender, years of professional experience, and other covariates where possible.

### Power Analysis

This pilot trial will enroll 44 providers and 140 adolescent and young adult patients over 6 months based on feasibility. Using a simple, parallel randomized design and accounting for providers evaluating more than 1 patient, we estimate the minimum detectable effect size (MDE) of 0.348. MDE is based on the following assumptions: α=.05, a 2-tailed test, with 80% power, and ICC of 0.01, 50% of variance in adolescents and young adults, and 50% of variance in providers explained, and all other covariates of interest (eg, age and gender) were balanced by randomization of STIckER to providers and their adolescent and young adult patients. With our sample size of 140 participants, we have the power to detect a minimum of 35% higher likelihood of being tested for STIs in the intervention arm compared to usual care.

The ICC of 0.01 and the assumption that 50% of the variance would be attributable to provider- versus patient-level factors were based on conservative estimates. These assumptions are intended to ensure sufficient power while accounting for possible provider clustering.

### Ethical Considerations

This study was reviewed and approved by the Institutional Review Board (IRB) at Columbia University (Protocol #AAAU7485). Written informed consent was obtained from all adult participants. For participants aged between 14 and 17 years, a waiver of parental consent was granted by the IRB, and assent was obtained using a standardized script and study summary tailored for adolescent comprehension. Agreement to participate was confirmed verbally in the presence of 2 study staff (typically the research coordinator and the physician attending the ED) to ensure understanding and voluntariness. All data are deidentified for analysis to ensure participant confidentiality. Participants received a US $10 pay card as compensation for their time.

## Results

Enrollment began in September 2023 and was completed in July 2025, with 44 providers and 139 adolescent and young adult participants enrolled across adult and pediatric EDs. All participants’ follow-up and survey data collection are complete. Data cleaning and primary analyses are currently underway, with dissemination of final results anticipated in late 2025.

## Discussion

### Hypothesis

This protocol outlines an RCT to assess the preliminary efficacy and implementation of the STIckER app, a shared decision-making tool to support gonorrhea and chlamydia testing in adolescents and young adults in EDs. We anticipate that the intervention will lead to a higher proportion of adolescents and young adults receiving comprehensive genitourinary and extragenital STI testing compared to usual care, and that both patients and providers will find the tool acceptable, appropriate, and feasible to implement.

### Comparison With Previous Work

Previous studies have highlighted significant gaps in STI testing, particularly for extragenital sites, in both outpatient and ED settings [3,29,30]. While SDM tools have been shown to improve patient engagement and outcomes in other areas of emergency medicine, such as analgesic choice and evaluation of chest pain, few, if any, have focused on sexual health. STIckER addresses this gap by applying the SDM model to STI testing decisions in the ED [15-19,31], a critical access point for many underserved youths [5,32]. By centering the patient’s values and preferences, this tool has the potential to both improve care and reduce disparities in STI screening access.

Beyond sexual health, a broader body of work on mHealth in emergency settings demonstrates the feasibility and moderate effectiveness of digital interventions across diverse health domains. For example, randomized trials of text-message interventions have shown reductions in hazardous drinking among young adults [33], while culturally tailored apps and SMS programs improved blood pressure control among Black adults with hypertension [34] and increased asthma-related follow-up visits for children [35]. Interventions that were linguistically accessible and rooted in behavioral change theory were most successful, underscoring the potential of ED-based digital tools like STIckER to promote equitable, patient-centered screening.

### Strengths and Limitations

This study has several notable strengths. It is designed as an RCT, a robust methodological approach that supports internal validity. The STIckER app was co-developed with input from both AYA patients and ED providers, ensuring relevance and usability for both stakeholder groups [20]. The tool was implemented across both pediatric and adult EDs that serve racially and socioeconomically diverse populations, increasing the relevance of findings for underserved communities. Additionally, the intervention’s digital, QR-code-based format is low-cost, scalable, and adaptable to a range of ED workflows.

However, the study also has important limitations. The STIckER app is currently only available in English, which will restrict its accessibility for non-English–speaking patients. The intervention focuses exclusively on gonorrhea and chlamydia and does not address other STIs that require blood-based testing, such as HIV or syphilis. Furthermore, the study is being conducted at a single academic medical center, limiting generalizability to other settings such as community hospitals or urgent care centers.

Given the nature of the intervention, participants and providers could not be blinded, which may have introduced expectation bias. Participants may have been able to infer their assignment based on the version of the app they received, potentially influencing their perception of care or engagement with STI testing discussions. As with many digital health trials, we anticipate variability in user engagement and potential drop-off during app use, which may affect intervention exposure. The inclusion of multiple secondary and implementation outcomes also raises the potential for type I errors. These limitations will be carefully considered during the interpretation of study results.

In a routine clinical setting, providers may not receive individualized training, and research staff would not be available to support app use. The absence of structured onboarding may reduce uptake and effectiveness. Future implementation efforts should explore how STIckER performs in lower-touch models, such as through automated workflows, integration into electronic health record systems, or peer-led education. Additionally, STIckER requires access to a smartphone and internet connection, which may pose challenges for adolescents and young adults from socioeconomically disadvantaged backgrounds. Devices or WiFi were not provided during this pilot, and future implementation efforts will need to consider strategies to improve equitable access.

Finally, although STIckER may influence broader awareness and discussion of STI testing, this pilot is limited to evaluating gonorrhea and chlamydia outcomes. The potential spillover effect on HIV or syphilis testing is not measured in this study but represents an important area for future research.

### Future Directions

If the pilot demonstrates feasibility and preliminary efficacy, future studies will focus on scaling the intervention to a larger, multicenter trial. Planned enhancements may include multilingual functionality and expansion of the tool to address additional STIs and linkage to care pathways. The app’s SDM framework may also serve as a model for other youth-focused digital health interventions in the ED. If effective, we will also explore partnerships with health care systems and public health organizations to promote adoption and sustainability, including potential integration into EHR systems and staff training modules.

## Supplementary material

10.2196/67103 Checklist 1 CONSORT-eHealth V1.6 checklist.

10.2196/67103 Checklist 2 SPIRIT checklist.

## Abbreviations:

AIM: acceptability of intervention measure
CFIR: Consolidated Framework for Implementation Research
ED: emergency department
EMR: electronic medical record
FIM: feasibility of intervention measure
GLM: generalized linear model
HCP: health care provider
HCP-P: health care provider participant
IAM: intervention appropriateness measure
ICC: intraclass correlation coefficient
RCT: randomized controlled trial
SDM: shared decision-making
STI: sexually transmitted infection
STIckER: STI Check in the Emergency Room
